# Central nervous system manifestations in hereditary transthyretin amyloidosis: a cross-sectional study with 151 patients

**DOI:** 10.1007/s00415-026-14146-9

**Published:** 2026-09-20

**Authors:** Luísa Sousa, Teresa Coelho, Joana Fernandes, Márcio Cardoso, Cristina Alves, Ana Martins da Silva, Ana Paula Sousa, Miguel Mendonça Pinto, Rafael Maniés Pereira, Luís Ruano, Carolina Lemos, Inês Baldeiras, Carolina Reis, Ricardo Taipa

**Affiliations:** 1 Unidade Corino de Andrade, Unidade Local de Saúde de Santo António, Porto, Portugal; 2 Unit for Multidisciplinary Research in Biomedicine, School of Medicine and Biomedical Sciences, University of Porto, Porto, Portugal; 3 Laboratory for Integrative and Translational Research in Population Health, Porto, Portugal; 4 Unidade Local de Saúde de Entre o Douro e Vouga, Santa Maria da Feira, Portugal; 5 Portuguese Brain Bank, Department of Neuropathology, Unidade Local de Saúde de Santo António, Porto, Portugal; 6 Center for Disease Mechanisms Research, Faculdade de Medicina da Universidade de Lisboa, Lisbon, Portugal; 7 Centro Académico Clínico Egas Moniz Health Alliance, Aveiro, Portugal; 8 Departamento de Ciências Médicas, Universidade de Aveiro, Aveiro, Portugal; 9 Faculty of Medicine, University of Coimbra, Coimbra, Portugal; 10 Center for Innovative Biomedicine and Biotechnology, University of Coimbra, Coimbra, Portugal; 11 Faculty of Sciences and Technology, University of Coimbra, Coimbra, Portugal; 12 Present Address: Alexion, AstraZeneca Rare Disease, Barcelona, Spain

**Keywords:** ATTRv amyloidosis, Hereditary transthyretin amyloidosis, Transthyretin, Central nervous system, Cerebral amyloid angiopathy, TFNE

## Abstract

**Background:**

Central nervous system (CNS) dysfunction is increasingly recognized in hereditary transthyretin (ATTRv) amyloidosis. We aimed to characterize CNS manifestations and assess associations with clinical variables and plasma neurofilament light chain (NfL), using history of transient focal neurological episodes (TFNEs) as the main outcome.

**Methods:**

We conducted a cross-sectional study at Unidade Corino de Andrade in Porto (January 2021–April 2023), including patients with ATTRv amyloidosis caused by the V30M mutation. History of TFNEs, stroke, seizures, headaches and symptoms/signs of cranial nerve dysfunction were assessed through patient interview and neurological examination. Plasma NfL was analyzed cross-sectionally and longitudinally (0–6–12 months).

**Results:**

151 patients were included (56.3% male, median age 48 [IQR 44–53], with a median disease duration of 15 years [IQR 11–20]. Most had early-onset disease (94.7%). TFNE history occurred in 29.1%, headaches in 23.8%, seizures in 6.6% and stroke in 1.3%. Patients with past TFNEs had longer disease duration than those without. No other robust differences were identified. Longitudinally, pNfL levels showed an overall effect of time (*p*=0.013), driven by a decrease between 6 and 12 months, with no significant differences between baseline and either follow-up timepoint. There were no differences in pNfL trajectories according to TFNEs history (time-by-TFNE interaction, *p*=0.416).

**Conclusions:**

The high frequency of CNS symptoms in this sample supports including CNS assessment in routine follow-up of patients with ATTRv amyloidosis with longstanding disease. Further longitudinal studies are needed to clarify determinants of CNS involvement.

**Supplementary Information:**

The online version contains supplementary material available at https://doi.org/10.1007/s00415-026-14146-9.

## Introduction

Hereditary transthyretin (ATTRv) amyloidosis is an autosomal dominant, multisystemic disease caused by the deposition of transthyretin (TTR) primarily in the peripheral nervous system and heart, leading to a progressive sensorimotor and autonomic neuropathy and cardiomyopathy [1, 2]. It is a rare disease worldwide, with higher prevalence or incidence reported in certain regions, including Portugal, Japan, and Sweden [3]. It is estimated that more than 10,000 patients are affected by this condition globally [4]. Disease-modifying therapies including liver transplantation, TTR stabilizers and *TTR* gene silencing therapies have drastically changed the course of the disease by stabilizing or reducing hepatic TTR production [1, 2]. Meanwhile, TTR is also synthesized by the choroid plexus and secreted into the CSF [5]. This intrathecal TTR production does not seem meaningfully affected, in theory, by current therapies, which exclusively target liver-derived TTR [6]. In the CNS, TTR progressively accumulates in the leptomeningeal membranes and arteries resulting in a superficial cerebral amyloid angiopathy [7–9]. Amyloid deposition is particularly prominent around the brainstem and spinal cord, especially in the early stages of disease [7]. It can be detected on amyloid-binding PET scans, with positive scans more likely in patients with longstanding disease and those with early-onset phenotypes, in whom infratentorial involvement is preferential [10, 11]. Leptomeningeal enhancement may also be seen on brain MRI, likely reflecting secondary disruption of the blood–brain barrier, and is also more prominent in the infratentorial region [12].

Improved survival brought by disease-modifying treatments has paradoxically led to higher prevalence of CNS dysfunction. Clinically, patients present transient focal neurological episodes (TFNEs), cerebral hemorrhages and cognitive dysfunction. TFNEs have been reported in up to 31% of liver-transplanted patients, usually in longstanding disease [13]. Most of the current understanding of CNS dysfunction in ATTRv amyloidosis comes from small cohorts of liver-transplanted patients with V30M (p.V50M) mutation. Symptoms such as headaches, seizures and cranial nerve dysfunction have been described in case reports, but their frequency is unknown [14]. Another urgent unmet need is the identification of accessible biomarkers for CNS involvement. These will be crucial in future clinical trials and clinical practice to help determine the onset and progression of CNS involvement. Blood neurofilament light chain (NfL), a marker of axonal damage, has been studied in several neurological diseases and has shown to differentiate asymptomatic carriers and controls from symptomatic ATTRv amyloidosis [15, 16]. However, its utility in detecting CNS involvement in ATTRv amyloidosis is unknown.

To broaden the spectrum of known CNS manifestations, we examined 151 patients with V30MATTRv amyloidosis for the presence of CNS symptoms and signs. The main outcome was a history of TFNE. We aimed to characterize the frequency and clinical features of CNS dysfunction and to identify potential associations with ATTRv amyloidosis-related factors, particularly disease duration and treatment status. Additionally, we explored whether plasma NfL (pNfL) is associated with a history of TFNEs.

## Methods

### Patient selection

A prospective observational cohort study with a cross-sectional primary analysis and an exploratory longitudinal pNfL analysis component was conducted at Unidade Corino de Andrade, one of the two referral centers for ATTRv amyloidosis in Portugal (following around 750 symptomatic patients), between January 2021 and April 2023, using a non-consecutive convenience sample. No formal a priori statistical sample-size calculation was performed. Eligible patients were identified during routine clinical practice and were invited to participate when physicians were available to perform study procedures. Due to clinical workload constraints, recruitment did not occur continuously, and inclusion was spread over the study period. Patients were eligible to participate if they met all of the following inclusion criteria: 1) age ≥18 years; 2) diagnosis of symptomatic ATTRv amyloidosis confirmed by a Neurologist at Unidade Corino de Andrade; 3) confirmed V30M mutation; and 4) provision of written informed consent. Patients were excluded if they had any of the following: 1) history of illiteracy; 2) developmental delay; or 3) major concurrent central nervous system disease (e.g., brain tumor or severe traumatic brain injury). Patients were enrolled at the time of the Neurology consultation.

### Clinical evaluation

Clinical evaluation was performed only at baseline for all patients, and CNS outcomes were analyzed as patient characteristics/history at inclusion rather than as prospectively observed incident events. Data collection comprised a semi-structured clinical telephone interview, a structured neurological examination including the ‘brainstem’ section of the Expanded Disability Status Scale (EDSS), used for multiple sclerosis [17], the Mini-Mental State Examination (MMSE), the Cognitive Failures Questionnaire (CFQ) [18], the Hospital Anxiety and Depression Scale (HADS), olfactory testing using the Brief Smell Identification Test (BSIT) [19] and a retrospective review of medical records. The structured neurological examination including the EDSS subscale was performed at the time of inclusion by one neurologist (LS, TC, MC, CA, AMS, APS or MMP). The remaining scores and tests were applied by a single investigator (JF) also at the time of inclusion. The review of medical records and clinical interview was performed by one neurologist (LS) in the days following inclusion. We defined 5 main CNS manifestations: 1) TFNEs, defined as episodes of self-limited neurological symptoms suggestive of cortical dysfunction as previously described [13, 20]. Briefly, symptoms classified as TFNE included transient language dysfunction (altered speech production with paraphasias), sensory symptoms (paresthesias or numbness affecting a hemibody, brachiofacial region, hemifacial region or affecting the entire limb from the shoulder or hip distally), visual disturbances (scintillating scotoma or hemifield visual loss) and motor manifestations (motor deficit or jerking movements affecting a hemibody, brachiofacial region, hemifacial region or affecting the entire limb from the shoulder or hip distally). TFNEs were further classified as positive (productive symptoms, associated with ‘gain of function’) or negative (associated with ‘loss of function’) [20]; 2) ischemic or hemorrhagic symptomatic stroke or transient ischemic attack (TIA); transient focal negative symptoms were classified as TIA if they were a single episode associated with a clear stroke etiology (atrial fibrillation, major intra or extracranial stenosis) or if associated with an acute vascular lesion in a compatible region in a brain CT/MRI; otherwise, episodes were classified as TFNEs; 3) headaches, if present consistently at least 2x/month, excluding headaches that started *before* the onset of ATTRv amyloidosis; 4) seizures, excluding symptomatic seizures and seizures in the context of a previously diagnosed epilepsy (before ATTRv amyloidosis onset); and 5) symptoms or signs compatible with brainstem or cranial nerve dysfunction not attributable to other causes. Variables related to demographic and ATTRv amyloidosis were collected through the interview and retrospective review of the medical records. Brain MRI was performed in a subset of patients (n=16) and the results have been previously reported [21]. An additional 13 brain MRIs were retrospectively reviewed as part of the same study [21]. Furthermore, ten patients (6.6%) included in the present cohort were previously described in an earlier study, from 2015, examining the frequency of focal neurological episodes [13]. The remaining patients have not been reported previously.

### pNfL measurement

An additional, exploratory, longitudinal analysis was performed, measuring pNfL prospectively at 3 timepoints (baseline, 6 and 12 months). Blood was collected into EDTA tubes, centrifuged at 2000 g for 10 min at 4 ºC and stored at −80 ºC in polypropylene aliquots. pNfL levels were determined by Single Molecule Array (SiMoA) in an SR-X platform (Quanterix, Lexington, MA), using the Nf-Light Advantage PLUS Kit and following manufacturer’s instructions. All specimens were assayed in duplicate between April and June and October of 2025, using the same batch of reagents (504,405).

### Statistical analysis

Categorical variables were summarized as counts and percentages. Given the marked asymmetry and the presence of outlying values in several variables, particularly pNfL, continuous data were generally described using medians and interquartile ranges. EDSS scores were dichotomized as normal/abnormal. Group comparisons between patients with and without history of TFNEs were performed using chi-square test, Fisher’s exact test and Mann–Whitney U test. Disease duration was analyzed primarily as a stratification factor rather than as a conventional explanatory covariate. To improve interpretability in selected exploratory analyses, an additional binary duration variable (≤ 15 vs >15 years) was created. Current disease-modifying treatment at study inclusion was analyzed in grouped categories. Due to sample size, patients treated with patisiran or inotersen were grouped under ‘gene silencing therapies’. Exploratory binary logistic regression analyses were performed with history of TFNEs as the dependent variable, and were retained only as secondary descriptive-adjusted analysis. The primary exploratory model included dichotomized disease duration, age at inclusion and treatment group. A secondary sensitivity model additionally included sex, NIS-LL and functional stage.

pNfL was analyzed cross-sectionally and longitudinally. Baseline pNfL levels were compared between patients with and without history of TFNEs using non-parametric methods. For longitudinal analysis across baseline, 6 and 12 months, a linear mixed-effects model was fitted using log-transformed pNfL values. Time, TFNE history, and their interaction were included to assess differences in pNfL trajectories according to TFNE history. A patient-specific random intercept was included, and a parsimonious covariance structure was selected to ensure model convergence. Missing data were not imputed, and each analysis used all available observations for the variables involved. All core analyses were performed using IBM SPSS Statistics, 31.0.0.0. All tests were two-sided. Results were interpreted at a 95% confidence level, with *p*<0.05 considered statistically significant.

## Results

### Sample characteristics and frequency of main CNS outcomes

One-hundred-and-fifty-two patients were initially included in the study. One of the patients subsequently withdrew consent to use their data and was removed from the analysis. The characteristics of the remaining 151 patients are displayed in Table 1. Median age was 48 years [44–53] and median disease duration was 15 years [11–20]. Most patients had early onset disease (94.7%, onset <age 50). A history of TFNEs was reported by 44/151 patients (29.1%), whereas headaches history was recorded in 36/151 (23.8%), seizures in 10/151 (6.6%) and stroke in 2/151 (1.3%) (Table 2). Reported headache onset occurred at a median disease duration of 10.5 years, while the first reported TFNE occurred at 15.5 years and seizures at 22.5 years.

**Table 1:** Sample characteristics

	All patients (*n*=151)
Age, median [P25-P75], years	48 [44–53]
Education, median [P25-P75], years	9 [6–12]
Female sex, *n* (%)	66 (43.7)
ATTRv amyloidosis duration, median [P25-P75], years	15 [11–20]
ATTRv amyloidosis age of onset, median [P25-P75], years	31 [26–37]
NIS-LL—median [P25-P75], valid *n*=132	14 [6–40.25]
mPND score—*n* (%)	
0, no sensory disturbances in feet	9 (6)
I, sensory disturbance in feet with preserved walking capacity	81 (53.6)
II, difficulties walking, but can walk without aid	40 (26.5)
IIIa, requires 1 stick/crutch to walk	12 (7.9)
IIIb, requires 2 sticks/crutches to walk	6 (4)
IV, uses a wheelchair/bedridden	3 (2)
Current treatment	
No treatment, *n*	1 (0.7)
Liver transplantation, *n* (%)	84 (55.6)
Tafamidis, *n* (%)	44 (29.1)
Patisiran, *n* (%)	14 (9.3)
Inotersen, *n* (%)	8 (5.3)
Creatinine, median [P25-P75], mg/dL, valid *n* =126	0.87 [0.71−1.03]
Baseline pNfL, median [P25-P75], pg/mL, valid *n* =137	20.24 [12.75−33.94]

*ATTRv* hereditary transthyretin; *NIS-LL* Neuropathy Impairment Score in the Lower Limbs; mPND score: modified polyneuropathy disability score; *pNfL* plasma neurofilament light chain

**Table 2:** CNS symptoms (patient reported) and signs (observed by neurologist)

	All patients (*n*=151)
**CNS symptoms**	
TFNEs, *n* (%)	44 (29.1)
Ischemic stroke, *n* (%)	2 (1.3)
Transient ischemic attack, *n* (%)	0
Hemorrhagic stroke, *n* (%)	0
Cranial nerve dysfunction symptoms, n (%)	60 (39.7)
Hyposmia, valid *n* = 141	32 (22.7)
Diplopia, valid *n* = 148	3 (2.0)
Hypoacusis, valid *n* = 150	28 (18.6)
Tinnitus, valid *n* = 150	15 (10.0)
Vertigo, valid* n* = 150	2 (1.3)
Facial sensory symptoms, valid *n* = 150	0
Dysphagia	1 (0.7)
Headaches, *n* (%)	36 (23.8)
Seizures, *n* (%)	10 (6.6)
**CNS signs (bedside neurological examination)**	
Palmar reflex—*n* (%)	0
Palmomentonian reflex—*n* (%)	8 (5.3)
Glabellar reflex—*n* (%)	5 (3.3)
Hand apraxia—*n* (%)	5 (3.3)
Orofacial apraxia—*n* (%)	0
Extraocular movements impairment (EDSS)—*n* (%)	1 (0.7)
Nystagmus (EDSS)—*n* (%)	18 (11.9)
Trigeminal damage (EDSS)—*n* (%)	3 (2.0)
Facial weakness (EDSS)—*n* (%)	3 (2.0)
Hearing loss (EDSS)—*n* (%)	37 (24.5)
Dysarthria (EDSS)—*n* (%)	5 (3.3)
Dysphagia (EDSS)—*n* (%)	3 (2.0)
Face fasciculations—*n* (%)	10 (6.6)
Tongue fasciculations—*n* (%)	8 (5.3)
Tongue atrophy—*n* (%)	6 (4.0)
Hyperreflexia—*n* (%)	5 (3.3)
Babinski sign—*n* (%)	3 (2.0)
Extrapyramidal signs—*n* (%)	13 (8.6)
Upper limb ataxia—*n* (%)	2 (1.3)
**Tests and questionnaires**	
MMSE, median [P25-P75]	29 [28–30]
CFQ, median [P25-P75]	25 [17–34]
HADS, median [P25-P75]	10 [7–15]
BSIT, median (IQR), valid *n*=147	10 [9–11]

*TFNEs* transient focal neurological episodes; *EDSS* Expanded Disability Status Scale; *MMSE* Mini-Mental State examination; *CFQ* Cognitive Failures Questionnaire; *HADS* Hospital Anxiety and Depression Scale; *BSIT* Brief Smell Identification Test

### Differences between patients with and without history of TFNE

Patients with a history of TFNEs reported substantially longer disease duration at inclusion than those without TFNEs (21(6) vs 14(6) years, *p*<0.001) (Table 3). By contrast, age did not differ materially between groups. No clear between-group differences were identified for NIS-LL or baseline pNfL. Serum creatinine was slightly higher in the TFNE group (0.97(0.41) vs 0.85(0.32) mg/dL, *p*=0.046), with a small absolute difference. Unadjusted categorical comparisons similarly did not show clear evidence of association for sex or Coutinho disease stage.

**Table 3:** Comparison of patients with and without history of TFNE

	No TFNE history (*n*=107)	With TFNE history (*n*=44)	*p*
Age, median (IQR), years	48 (13)	49 (7)	0.327
Sex, n of females (%)	51 (47.7)	15 (34.1)	0.127
ATTRv amyloidosis duration, median (IQR), years	14 (6)	21 (6)	<0.001
Coutinho stage			0.891
Stage 1 (fully ambulant) *n* (%)	89 (83.2)	37 (84.1)	–
Stage 2 (need of walking aids), *n* (%)	18 (16.8)	7 (15.9)	–
NIS-LL, median (IQR)	12 (35)	18 (36.3)	0.354
Current treatment*			<0.001*
Liver transplantation, *n* (%)	45 (42.5)	39 (88.6)	–
Tafamidis, *n* (%)	42 (39.6)	2 (4.5)	–
Gene silencing therapies,* n* (%)	19 (17.9)	3 (6.8)	–
Vascular risk factors			
Hypertension, *n* (%)	24 (22.4)	4 (9.1)	0.066
Diabetes Mellitus, *n* (%)	3 (2.8)	0	
Dyslipidemia, *n* (%)	24 (22.4)	7 (15.9)	0.506
Antiplatelet therapy *n* (%)	17 (15.9)	6 (13.6)	0.808
Anticoagulant therapy, *n* (%)	4 (3.8)	0	
Creatinine, median (IQR), mg/dL	0.85 (0.3)	0.97 (0.4)	0.046
Baseline NfL, median (IQR), pg/ml	21.34 (25.8)	20.54 (17.9)	0.478

*Treatment-category comparison was calculated after exclusion of the only patient without disease-modifying therapy. The unadjusted difference in treatment distribution between TFNE-history groups should be interpreted cautiously, given the potential confounding by disease duration and therapeutic era. Analyses were performed using available cases for each variable

### TFNE history according to disease duration

**Fig. 1:**
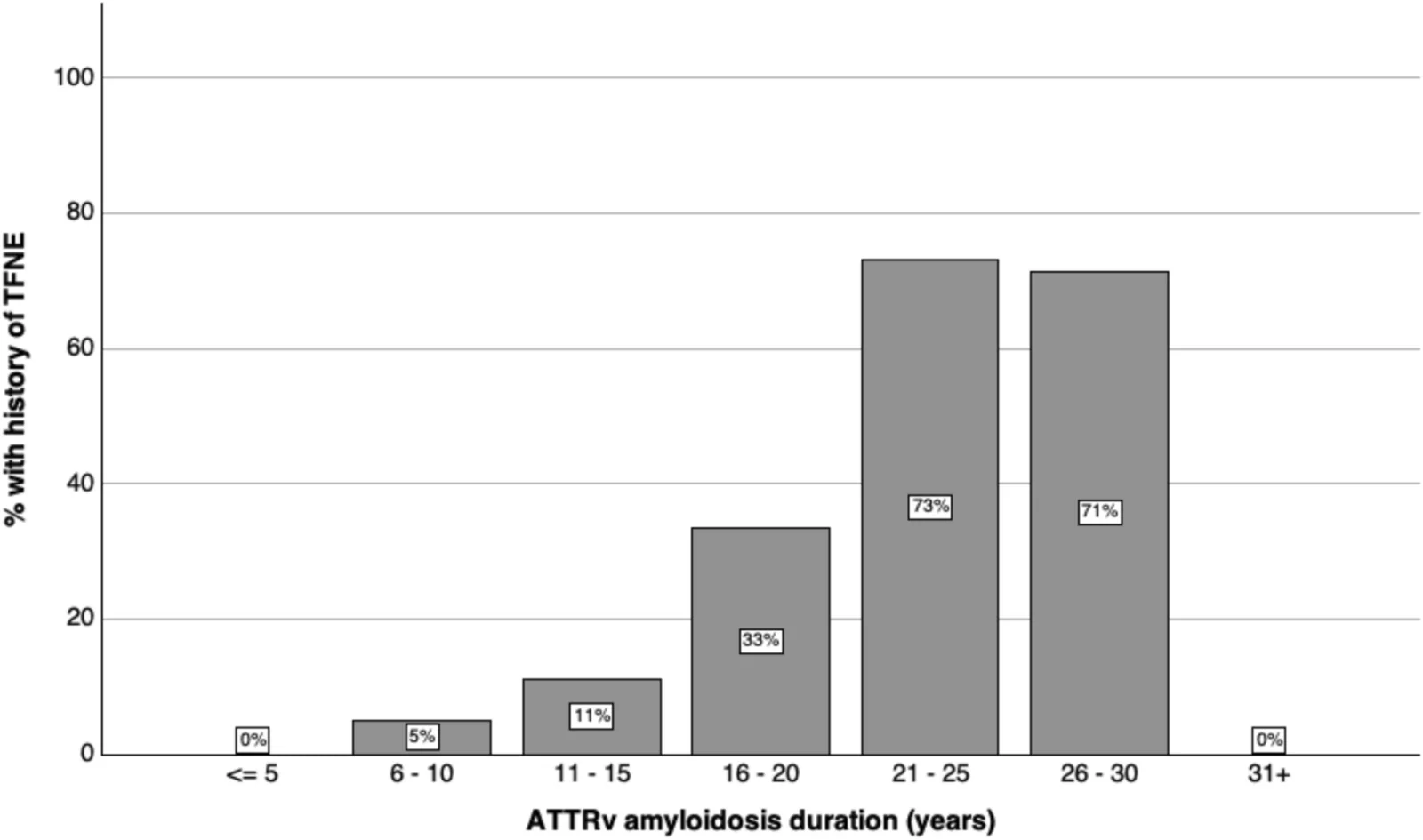
History of TFNE by disease duration categories—percentage of patients with a history of TFNE in each disease-duration category. The chart describes the association between two patient characteristics at the time of inclusion; it does not represent incidence or the longitudinal evolution of the event over time

The proportion of patients with a history of TFNEs was higher in longer disease-duration strata, ranging from 0% in patients with ≤5 years of disease to 71.4% in the 26–30-year strata (Fig. 1). When disease duration was dichotomized, TFNE history was present in 6/76 (7.9%) patients with ≤15 years of disease and in 38/75 (50.7%) patients with >15 years of disease.

### TFNE history and disease-modifying therapy

In unadjusted analyses, current treatment distribution differed between patients with and without TFNE history (Table 3). Among patients with a history of TFNEs, 39/44 (88.6%) were liver transplant recipients, compared with 45/106 (42.5%) among those without TFNEs. However, treatment allocation was strongly dependent on disease duration. For patients with ≤15 years of disease, treatment distribution was 26.7% transplantation, 49.3% tafamidis, and 24.0% gene silencing therapies; for >15 years, it was 85.3%, 9.3%, and 5.3%, respectively (Online Resource 1). When analyzing the distribution of treatment across duration strata and history of TFNE (Table 4), the association between treatment and TFNE weakened substantially. This suggests that the raw difference observed in treatment groups between TFNE groups is mostly explained by disease duration.

**Table 4:** History of TFNE according to treatment category, stratified by disease duration

Disease duration	TFNE history group	Liver transplant	Tafamidis	Gene silencing therapies
≤15 years	No TFNE history (*n*=69)	16	36	17
	With TFNE history (*n*=6)	4	1	1
>15 years	No TFNE history (*n*=37)	29	6	2
	With TFNE history (*n*=38)	35	1	2

Frequencies of patients in each treatment category according to disease duration and TFNE history. The only untreated patient was excluded from this table. The crude association between treatment category and TFNE history was attenuated after stratification by disease duration, supporting the interpretation that treatment comparisons are substantially confounded by disease duration and therapeutic era

In the primary exploratory logistic model including dichotomized disease duration, age and current treatment, disease duration >15 years was associated with higher odds of a history of TFNEs (OR 5.85, 95%CI 1.98–17.29; *p*<0.001) (Online Resource 2). Compared with liver transplantation, tafamidis was associated with lower odds of reporting a history of TFNEs (OR 0.13, 95%CI 0.03–0.64; *p*=0.012), whereas gene silencing therapies were not significantly associated (OR 0.44, 95%CI 0.10–1.91; *p*=0.274). In the sensitivity model additionally including sex, NIS-LL and functional stage, disease duration >15 years remained associated with TFNE history (OR 10.72, 95%CI 2.84–40.52; *p*<0.001, Online Resource 2), while treatment category, sex, NIS-LL and functional stage were not statistically significant.

### TFNE characteristics

Among the 44 patients with a history of TFNE, most patients had episodes of language dysfunction (35/44, 79.5%), followed by negative and positive sensory symptoms (Online Resource 3). When patients were asked to indicate their predominant recurrent symptom, sensory symptoms were the most frequently cited (28/44, 63.6%). Most patients experienced more than one type of TFNE (24/44, 55%). When asked whether their most common TFNE was positive or negative, 25 patients (56.8%) reported predominantly negative symptoms. The median number of total episodes of TFNEs per patient was 10 [12]. The median duration of the episodes was 15 min (22,5). Six patients reported TFNEs with ≥1 h and maximum duration was 12 h. Some patients reported headaches in at least half of their episodes (17/44, 38.6%). Patients with reported associated headache had longer TFNE (30[120] vs 15[25], *p*=0.01). Among patients with TFNE, men had more frequent episodes than women (11[12] vs 6[8], *p*=0.05). The number of different TFNEs each patient had, total number of TFNEs or TFNE duration did not correlate with disease duration.

Among patients with TFNE, 24 (54.6%) underwent an EEG at our center. Five patients had abnormal results: four EEGs demonstrated focal slowing, three showed paroxysmal slow-wave activity, and none were reported to have epileptiform discharges. Twenty patients (19.6%) were treated with antiepileptic drugs, regardless of motive. Patients under antiepileptic drugs had more frequent TFNEs (12[14] vs 5.5[9], *p*=0.03).

### Other CNS outcomes

Symptomatic ischemic stroke was experienced by two patients. One was a patient with 19 years of disease duration treated with liver transplantation who had a cerebellar stroke of undetermined source. The other patient had 8 years of disease duration and was receiving patisiran when they developed a large-vessel ischemic stroke after pulmonary embolism, attributed to an atrial septal aneurysm and patent foramen ovale. No patients had TIA, clinically evident intracerebral or subarachnoid hemorrhages.

Symptoms related to cranial nerve dysfunction were reported by sixty patients (39.7%) (Table 2). The most common was hyposmia (22.7%) followed by hypoacusis (18.6%) and tinnitus (10%). Hypoacusis was unilateral in 8/28 of patients. On the bedside neurological examination, the most common objective finding was hearing loss (24.5%), followed by impaired odor identification (BSIT score ≤8, 13.9%) and nystagmus (Table 2). Patients with hearing loss were older (53[11] vs 47[8], *p*<0.001) and had longer ATTRv amyloidosis duration (19[11] vs 15[7], *p*=0.02).

Headaches (at least 2 per month) were reported by 36 patients (23.8%). Patients with headache history had longer disease duration than those without headaches (18[6] vs 15[8], *p*=0.003). Headache frequency data were available for 24 patients, 7 (29.2%) of whom had ≥15 episodes/month. Headache frequency or duration did not correlate with disease duration. The median frequency of headaches per month was 4[5]. Most patients (22/36, 61.1%) had migraine according to ICHD3 criteria. Median duration of headache was 12 h (720 min [1320]).

Seizure history was reported by ten patients (6.6%), all treated with liver transplantation. Patients with seizure history reported longer disease duration at inclusion than those without seizures (22.5[6] vs 15[8], *p*=0.002). All patients had either a single seizure or multiple seizures in a single day.

### Plasma neurofilament light chain

Median baseline pNfL in the overall sample was 20.24 pg/mL [12.75–33.94]. Baseline pNfL did not differ between patients with and without TFNE history (20.54 (17.89) vs 21.34 (25.76) pg/mL, *p*=0.478). Baseline pNfL showed exploratory correlations with NIS-LL (r(118)=0.546, *p*<0.001) and creatinine levels (r(110)=0.332, *p*<0.001), but not with disease duration. Given the skewed distribution of pNfL, these associations should be interpreted cautiously and were considered contextual biomarker findings.

### *Longitudinal pNfL analysis*

**Fig. 2:**
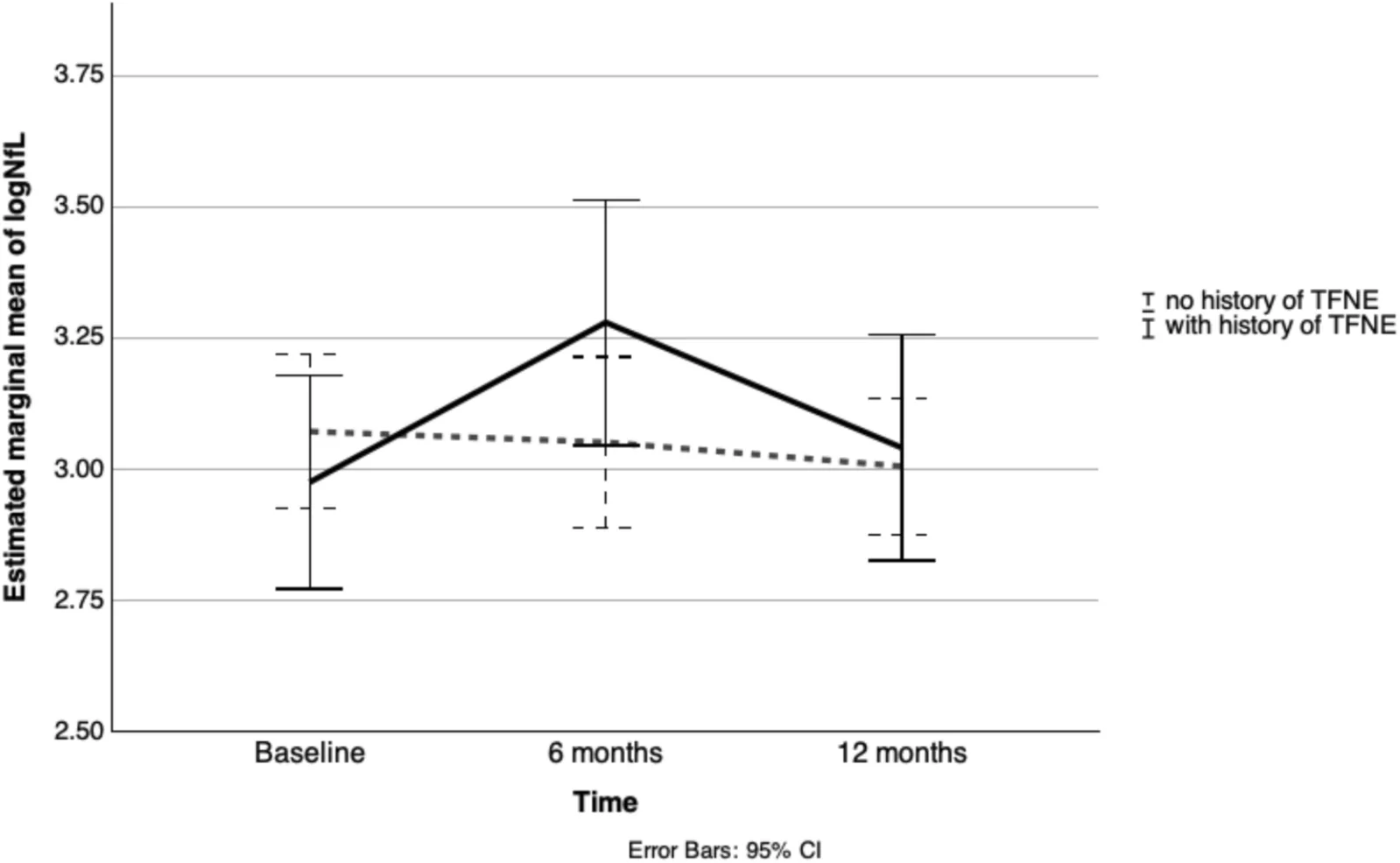
Estimated marginal means from the mixed model for logNfL at baseline, 6 months, and 12 months, stratified by history of TFNEs. A global effect of time is observed, with no main effect of the TFNE group and no time × TFNE interaction

In the longitudinal mixed-effects model using log-transformed pNfL, there was evidence of an overall time effect (*F*=4.457, *p*=0.013), without a main effect of TFNE history (F=0.007, *p*=0.935) nor a time-by-TFNE interaction (*F*=0.883, *p*=0.416) (Online Resource 4). Pairwise comparisons showed evidence of a difference only between 6 and 12 months (difference 0.102, *p*=0.003), with no significant differences between baseline and 6 months or between baseline and 12 months. Thus, although pNfL levels changed over time overall, the absence of a time-by-TFNE interaction indicated that these temporal changes did not differ according to TFNE history (Fig. 2).

## Discussion

In this large cohort of patients with V30MATTRv amyloidosis, CNS symptoms were very common, particularly among those with more than 15 years of disease duration. Notably, CNS symptoms were more heterogeneous than previously recognized, as prior studies largely focused on TFNEs as the primary manifestation [13, 22, 23]. The largest study to date in this population, published 11 years ago, included 87 liver transplant recipients and primarily evaluated focal neurological events [13]. Consistent with those findings, we observed a similar frequency of TFNEs, onset after approximately 15 years of systemic disease, and an association with longer disease duration [13]. Similar associations between disease duration and TFNEs frequency have also been reported by other investigators [22, 23]. Our study expands on previous works by including patients receiving a broader range of disease-modifying therapies beyond liver transplantation, assessing a wider spectrum of CNS symptoms and signs, and by evaluating the relationship between CNS symptoms and pNfL.

From all the CNS symptoms and signs analyzed, TFNEs seem to be the most useful core symptom to consider as a “clinical marker” of CNS dysfunction. TFNEs were the most common CNS manifestation overall and were more frequent in patients with longstanding disease. In fact, in this cohort, in individuals with more than 20 years of disease duration, more than 70% had a history of TFNE. As patient longevity increases, it is expected that, at least in early-onset V30M amyloidosis, most patients will have symptoms of CNS dysfunction [10]. This highlights the need for further research into CNS dysfunction in this population, particularly given the lack of approved therapies with demonstrated efficacy in the CNS [24, 25]. Importantly, among patients with a history of TFNEs, neither symptom diversity nor event frequency was associated with disease duration. This lack of association suggests that clinical criteria alone may have limited utility in reflecting the status of CNS involvement. Accordingly, these findings suggest that future biomarkers of CNS involvement will play a key role in guiding the development of new therapies.

As for the TFNE characteristics, they align with previous descriptions [13, 22, 23]: TFNEs, in general, were recurrent, mostly associated with transient loss of function and lasted less than 1 h. The fact that TFNEs were frequently associated with loss of function and lasted less than 24 h supports the empirical observation that these phenomena are clinically indistinguishable from transient ischemic attacks. This may present a challenge in clinical practice, particularly when a patient presents with a first event. Recurrence appears to be the main distinguishing feature between TFNEs and TIA, although recurrent episodes alone are insufficient to confidently exclude a cerebrovascular event [26]. A comprehensive stroke work-up, including brain MRI whenever possible, is advisable when patients first present with a TFNE. Decisions regarding primary or secondary stroke prevention are particularly challenging in this population, given the limited evidence on cerebral ischemic and hemorrhagic events, which is based primarily on case reports and small case series [13, 27]. Risk assessments are often extrapolated from other conditions, particularly cerebral amyloid angiopathy associated with amyloid-β (Aβ-CAA). In Aβ-CAA, the presence of TFNE is strongly associated with acute convexity subarachnoid hemorrhage or cortical superficial siderosis, which represent acute and chronic manifestations of bleeding at the cerebral surface, likely secondary to amyloid-related vessel involvement [20, 28, 29]. Additionally, TFNEs are associated with a high risk of subsequent lobar intracerebral hemorrhage or subarachnoid hemorrhage [20, 28]. Although the clinical features of TFNEs associated with both conditions are virtually indistinguishable, the underlying hemorrhagic risk may not be equivalent. Nonaneurysmal subarachnoid hemorrhage and lobar intracerebral hemorrhages have been described in ATTRv amyloidosis [5, 13, 30], but superficial siderosis does not appear to be common in neuropathological studies of patients with ATTRv amyloidosis [7, 9]. In a Portuguese neuropathological study, only 2/16 patients (12.5%) showed superficial siderosis, described as mild [7]. Notably, two patients from the same cohort had previous intracerebral hemorrhages, without superficial siderosis in other brain regions. Similarly, a previous brain MRI study including 5 patients with TFNEs found no underlying ischemic or hemorrhagic lesions, despite positive ^11^C-Pib retention on brain PET scans, consistent with amyloid deposition [22]. Another study showed normal MRIs in 5 patients with TFNEs, including patients with more than 20 episodes [8]. Accordingly, in a prospective and retrospective MRI study including patients from this cohort, no superficial siderosis or subarachnoid hemorrhages were found, including in 12 patients with TFNEs [21]. In contrast, in a French retrospective MRI study, 16% of patients had a history of clinical stroke and 31% met imaging criteria for CAA [31]. This cohort included a high proportion of patients with late onset disease or non-V30M mutations. These differences in stroke frequency may therefore reflect distinct phenotypes of CNS involvement, potentially influenced by age of onset and mutation type, as well as differences in inclusion criteria. Nevertheless, retrospectively, the presence of TFNEs, migraine or symptomatic stroke did not differ significantly between patients with or without CAA criteria [31].

In our cohort, stroke was a rare event, and only one patient had an ischemic event of undetermined source which could potentially be related to ATTRv. Previous neuropathological and MRI studies of Portuguese patients with the V30M mutation, including MRI data from some patients included in the present study, have likewise shown few symptomatic or asymptomatic ischemic vascular events [7, 21]. Conversely, in a retrospective Swedish cohort of patients with V30M ATTRv amyloidosis who underwent liver transplantation, ischemic stroke was observed in 16% of cases [27]. The cross-sectional design of our study and inclusion of only living patients introduce potential survival bias, as patients who experienced fatal strokes would not be represented in the cohort. Consequently, the observed frequency of stroke may underestimate the true incidence of stroke in this population. A better understanding of the epidemiology and risk factors for both ischemic and hemorrhagic events in this population is needed, particularly given their potential implications for clinical management. Larger studies using different study designs will be necessary to address this.

Importantly, the frequency of a history of TFNE and/or the frequency of episodes in patients with TFNE may have been modified in patients under antiepileptic treatment for either migraine, TFNEs or seizures. The effect of antiepileptics on TFNEs has never been assessed but they reduce the occurrence of focal seizures and cortical spreading depression, which may underlie at least some of these events [32]. Still, distinguishing the underlying cause of a TFNE (focal seizure, cortical spreading depression or other) may not be clinically essential; what remains to be determined is whether TFNE occurrence or frequency has prognostic implications or is directly related to the severity of CNS amyloid deposition. Longitudinal studies will be necessary to address these issues.

Apart from disease duration, no unequivocal associations with other clinical variables were identified. The unadjusted association between treatment with tafamidis and lower odds of having experienced TFNEs should be interpreted with caution, given substantial confounding by therapeutic era, disease duration, and potentially disease severity. Patients with longer disease duration, who may be at higher risk of TFNEs, were much more likely to have received liver transplant, reflecting its exclusive use in an earlier therapeutic period. Additionally, tafamidis is preferentially used in patients with less severe disease, raising the possibility of channeling bias. Because both disease duration and severity may be associated with TFNEs risk, these differences in treatment allocation may contribute to the observed association. Consistent with this interpretation, in a sensitivity analysis adjusting for sex, disease severity score, and functional stage, the association between treatment category and history of TFNE was no longer statistically significant.

Nevertheless, some patients receiving tafamidis or gene silencing therapies also show history of TFNEs. Despite the increasingly recognized CNS dysfunction in ATTRv amyloidosis, CNS-related outcomes have never been included in clinical trials and the effect of currently used therapies in the CNS is unknown. Tafamidis 20 mg/day has been found in the cerebrospinal fluid of patients with ATTRv amyloidosis, in a concentration allowing a moderate stabilization of TTR [33]. Yet, patients under tafamidis still display CNS symptoms and ^11^C-PiB uptake, a measure of amyloid deposition, in cerebral PET scans [8, 33]. Higher doses (tafamidis meglumine 80 mg or tafamidis 61 mg), approved for the treatment of ATTRv amyloidosis cardiomyopathy, have been shown, in vitro, to decrease wild-type TTR dissociation in CSF [34]. However, its effect on clinical manifestations has not been systematically evaluated. Similarly, although RNA interference and antisense oligonucleotide treatments are generally considered to have limited penetration of the blood–brain barrier, few case reports have described stabilization of CNS symptoms and improvement in leptomeningeal enhancement following treatment [35, 36]. Given the widespread use of these therapies in ATTRv amyloidosis and the paucity of systematic evidence, evaluating their effects in longitudinal studies and clinical trials represents an urgent unmet need.

Another important finding is that the variety of CNS symptoms patients can present goes well beyond TFNEs. Patients often have a history of headache, seizures and different symptoms that can be attributable to cranial nerve dysfunction. Patients with a history of seizures had longer disease durations than those without; it will be important to confirm in further studies if this is a symptom that increases with disease duration. Headache was a common symptom in this cohort, although the overall frequency of headache, including migraine-type headache was similar to that reported in the general population [37].

Regarding cranial nerve dysfunction, the most frequent symptom was hearing loss, which was reported by 18.5% of patients and found in the bedside neurological examination in 24.5% of cases. A previous study found hearing loss to be more common and severe than expected in late onset ATTRv amyloidosis [38]. Although potentially multifactorial, the predominance of sensorineural hearing loss in patients with the V30M mutation [38], together with the marked amyloid deposition around the brainstem and cranial nerve emergence these patients often present [7], suggests cranial nerve involvement may be a contributing mechanism. Hearing loss may be particularly relevant in this population because it is a known risk factor for cognitive decline, especially in patients who already have high risk of vision loss [39]. Importantly, the bedside hearing assessment used in this study does not constitute a comprehensive evaluation of hearing loss and does not permit definitive characterization of the underlying deficit. Rather, these findings should be considered screening observations that may help identify patients who warrant formal audiologic assessment. Additionally, hearing loss has a significant association with patient’s age, and it may not reflect a direct association with ATTRv amyloidosis pathophysiology.

Finally, in this study, baseline pNfL was not different between patients with and without history of TFNE. In the longitudinal analysis, pNfL levels changed over time overall, with a significant difference found only between 6 and 12 months. Notably, however, pNfL decreased during this interval, and no significant differences were observed between baseline and either follow-up time point. Furthermore, the trajectory did not differ according to TFNE history, as indicated by the absence of a time-by-TFNE interaction. NfL is a recognized marker of axonal injury in both the peripheral and central nervous systems [40]. In some previous studies in ATTRv amyloidosis, it is associated with clinical severity scores of peripheral nerve disease [41]. Consistent with this, we found an association between pNfL and NIS-LL. The contribution of peripheral nerve disease to circulating pNfL levels may be substantial and could limit the ability to detect a relatively small CNS-derived NfL signal associated with prior TFNE. It would be interesting, in this context, to evaluate whether NfL levels in cerebrospinal fluid may provide additional information regarding CNS axonal injury. Alternatively, axonal damage may not be a central factor in the pathophysiology of CNS involvement, although the mechanisms which underlie CNS dysfunction are largely unknown. Additionally, pNfL levels could vary depending on the recent occurrence of TFNEs, stroke or seizures, which was not accounted for in this study. Future studies will be necessary to assess NfL relevance in CNS involvement.

This study has several limitations. First, the use of a nonrandomized, convenience sample from a single center may have introduced selection bias and limited the generalizability of our findings. No formal a priori sample-size calculation was performed; rather, the sample size was determined pragmatically by the availability of eligible patients for assessment during the study period. In addition, patient recruitment depended on physician availability during routine practice, leading to intermittent and nonconsecutive inclusion over an extended period and potentially limiting representativeness. Although the observed TFNE history frequency was consistent with previous studies, suggesting no major distortion, selection bias should be considered when interpreting the findings. Second, self-reported symptoms carry the risk of recall bias; this risk was reduced by complementing patient reports with the information from medical records. Transient symptoms can be difficult to distinguish from symptoms of non-CNS origin; in cases of uncertainty, transient symptoms were not classified as TFNEs. Additionally, all transient focal symptoms of suspected cortical origin were classified as TFNEs, irrespective of the presumed underlying mechanism. Another important limitation is that only a small proportion of patients underwent brain MRI or EEG, consequently, some of these events may be focal seizures, migraine auras or TIAs. We considered that attempting to classify transient symptoms by etiologic categories based on clinical features alone would likely introduce greater misclassification.

Finally, this is a homogeneous population of patients of Portuguese descent, with V30M mutation and mostly an early-onset disease. Most publications describing CNS manifestations in patients with ATTRv amyloidosis have focused predominantly on patients with early-onset disease [10, 13, 23, 27]. To the best of our knowledge, no study has specifically reported the frequency of CNS manifestations in patients with late-onset disease. Nevertheless, these patients also experience TFNEs or other CNS symptoms [8, 31]. Furthermore, several other non-V30M mutations are associated with CNS phenotypes that are particularly severe [14]. The findings of our study may not be generalizable, therefore, to patients with late-onset disease or other mutations known to affect the CNS.

The high frequency of CNS symptoms in this sample suggests that routine follow-up of patients with longstanding disease should include CNS assessment. Larger, longitudinal studies will be essential to clarify the temporal course and determinants of CNS dysfunction and to evaluate associations between currently used therapies and clinical outcomes.

## Supplementary Information

Below is the link to the electronic supplementary material.

Supplementary file1 (DOCX 23 KB)

## Data Availability

The dataset used during the current study is not publicly available due to restrictions related to participant privacy and the terms of informed consent. De-identified data may be made available to qualified researchers upon reasonable request to the corresponding author, subject to appropriate ethical and institutional approvals.
