# Supplementary material for: Central nervous system manifestations in hereditary transthyretin amyloidosis: a cross-sectional study with 151 patients
Source: J Neurol. 2026 Sep 20;273(10):611. doi: 10.1007/s00415-026-14146-9 (PMC13590438; doi:10.1007/s00415-026-14146-9)
Supplement: Supplementary file 1 [file 415_2026_14146_MOESM1_ESM.docx]

**SUPPLEMENTARY DATA**

**Central nervous system manifestations in hereditary transthyretin amyloidosis: a cross-sectional study with 151 patients**

Journal of Neurology

Luísa Sousa, Teresa Coelho, Joana Fernandes, Márcio Cardoso, Cristina Alves, Ana Martins da Silva, Ana Paula Sousa, Miguel Mendonça Pinto, Rafael Maniés Pereira, Luís Ruano, Carolina Lemos, Inês Baldeiras, Carolina Reis, Ricardo Taipa

Corresponding Author: Luísa Sousa, [luisacmsousa@gmail.com](mailto:luisacmsousa@gmail.com), Unidade Corino de Andrade, Centro Hospitalar Universitário de Santo António, Porto, Portugal

**Table of contents**

Online Resource 1 – Distribution of treatment according to disease duration

Online Resource 2 - Exploratory logistic regression for TFNE history

Online Resource 3 - Symptoms reported by patients during TFNEs

Online Resource 4 - Baseline NfL and mixed effects model using logNfL

**Online Resource table 1 – Distribution of treatment according to disease duration**

| **Disease duration category** | **Liver transplant** | **Tafamidis** | **Gene silencing therapies** |
| --- | --- | --- | --- |
| **≤15 years (n=75)** | 20 (26.7%) | 37 (49.3%) | 18 (24.0%) |
| **>15 years (n=75)** | 64 (85.3%) | 7 (9.3%) | 4 (5.3%) |

This table directly documents structural confounding between treatment and disease duration, which limits any strong interpretation of the crude treatment comparisons.

**Online Resource table 2 – Exploratory adjusted logistic regression for TFNE history**

| **Variable** | **OR** | **95% CI** | **p** |
| --- | --- | --- | --- |
| **Primary exploratory logistic model (n=150)** | | | |
| Disease duration >15 years (vs ≤15 years) | 5.85 | 1.98–17.29 | **<0.001** |
| Tafamidis (vs liver transplant) | 0.13 | 0.03-0.64 | **0.012** |
| Gene silencing therapies (vs liver transplant) | 0.44 | 0.10–1.91 | 0.274 |
| Age (by year) | 1.01 | 0.95-1.07 | 0.838 |
| **Sensitivity model (n=131)** | | | |
| Disease duration >15 years | 10.72 | 2.84-40.52 | **<0.001** |

Note: these models are exploratory and subordinate to the primary analysis based on stratification by disease duration. They should not be interpreted as predictive or causal models. Other covariates included in the sensitivity model, namely treatment category, sex, NIS-LL, functional stage and age, were not statistically significant.

**Online Resource table 3 – Symptoms reported by patients during TFNEs**

|  | **Patients with history of TFNEs (n=44)** |
| --- | --- |
| Language dysfunction - n (%) | 35 (79.5) |
| Sensory symptoms, positive - n (%) | 16 (36.4) |
| Sensory symptoms, negative - n (%) | 17 (38.6) |
| Visual symptoms, positive - n (%) | 10 (22.7) |
| Visual symptoms, negative - n (%) | 5 (11.4) |
| Motor symptoms, positive - n (%) | 3 (6.8) |
| Motor symptoms, negative - n (%) | 13 (29.5) |

TFNEs: transient focal neurological episodes. Proportion of patients reporting at least one episode of transient neurological dysfunction with these characteristics. *Positive* symptoms refer to phenomena associated with ‘gain of function’ such as paraesthesia, scintillating scotoma or jerking movements, whereas *negative* symptoms refer to loss of function, including numbness, hemifield visual loss or motor deficit.

**Online Resource table 4 – Baseline NfL and mixed effects model using logNfL**

| **Analysis component** | **Result** | **Statistical inference** |
| --- | --- | --- |
| **Baseline NfL, median (IQR), pg/mL** | No TFNE history: 21.34 (25.76)  With TFNE history: 20.54 (17.89) | Mann-Whitney U test, p=0.478 |
| **Overall time effect (mixed-effects model using logNfL)** | F=4.457 | **p=0.013** |
| **Main effect of TFNE history** | F=0.007 | p=0.935 |
| **Time by TFNE interaction** | F=0.883 | p=0.416 |
| **Estimated marginal means for logNfL** | Baseline 3.049  6 months 3.111  12 months 3.009 | - |
| **Pairwise comparisons** | - | - |
|  | Baseline vs 6 months: | p=0.081 |
|  | Baseline vs 12 months: | p=0.224 |
|  | 6 months vs 12 months | **p=0.003** |
| **Estimated marginal means by TFNE history** | No TFNE history: 3.051  With TFNE history: 3.061 | p=0.935 |
